# 2-(3-Methyl­sulfanyl-5-propyl-1-benzofuran-2-yl)acetic acid

**DOI:** 10.1107/S1600536809012124

**Published:** 2009-04-08

**Authors:** Hong Dae Choi, Pil Ja Seo, Byeng Wha Son, Uk Lee

**Affiliations:** a Department of Chemistry, Dongeui University, San 24 Kaya-dong, Busanjin-gu, Busan 614-714, Republic of Korea; b Department of Chemistry, Pukyong National University, 599-1 Daeyeon 3-dong, Nam-gu, Busan 608-737, Republic of Korea

## Abstract

The title compound, C_14_H_16_O_3_S, was prepared by alkaline hydrolysis of ethyl 2-(3-methyl­sulfanyl-5-propyl-1-benzofuran-2-yl)acetate. In the crystal structure, the carboxyl groups are involved in inter­molecular O—H⋯O hydrogen bonds, which link the mol­ecules into centrosymmetric dimers. These dimers are further packed into stacks along the *a* axis by weak C—H⋯π inter­actions.

## Related literature

For the crystal structures of similar 2-(3-methyl­sulfanyl-1-benzofuran-2-yl) acetic acid derivatives, see: Seo *et al.* (2007 ▶); Choi *et al.* (2008 ▶).
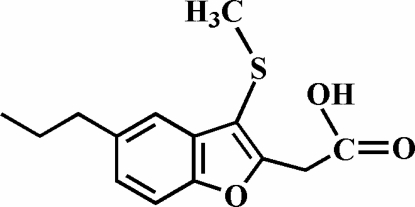


## Experimental

### 

#### Crystal data

C_14_H_16_O_3_S
*M* *_r_* = 264.33
Triclinic, 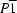

*a* = 5.1727 (6) Å
*b* = 8.173 (1) Å
*c* = 16.614 (2) Å
α = 94.321 (2)°
β = 95.831 (2)°
γ = 91.110 (2)°
*V* = 696.50 (14) Å^3^
*Z* = 2
Mo *K*α radiation
μ = 0.23 mm^−1^
*T* = 298 K
0.20 × 0.20 × 0.05 mm

#### Data collection

Bruker SMART CCD diffractometer
Absorption correction: none
3658 measured reflections
2389 independent reflections
1425 reflections with *I* > 2σ(*I*)
*R*_int_ = 0.049

#### Refinement

*R*[*F* ^2^ > 2σ(*F* ^2^)] = 0.059
*wR*(*F* ^2^) = 0.151
*S* = 1.06
2389 reflections
165 parameters
H-atom parameters constrained
Δρ_max_ = 0.21 e Å^−3^
Δρ_min_ = −0.24 e Å^−3^

### 

Data collection: *SMART* (Bruker, 2001 ▶); cell refinement: *SAINT* (Bruker, 2001 ▶); data reduction: *SAINT*; program(s) used to solve structure: *SHELXS97* (Sheldrick, 2008 ▶); program(s) used to refine structure: *SHELXL97* (Sheldrick, 2008 ▶); molecular graphics: *ORTEP-3* (Farrugia, 1997 ▶) and *DIAMOND* (Brandenburg, 1998 ▶); software used to prepare material for publication: *SHELXL97*.

## Supplementary Material

Crystal structure: contains datablocks global, I. DOI: 10.1107/S1600536809012124/zl2191sup1.cif

Structure factors: contains datablocks I. DOI: 10.1107/S1600536809012124/zl2191Isup2.hkl

Additional supplementary materials:  crystallographic information; 3D view; checkCIF report

## Figures and Tables

**Table 1:** Hydrogen-bond geometry (Å, °)

*D*—H⋯*A*	*D*—H	H⋯*A*	*D*⋯*A*	*D*—H⋯*A*
O2—H2*O*⋯O3^i^	0.82	1.86	2.679 (3)	174
C12—H12*A*⋯*Cg*^ii^	0.97	3.04	3.770 (3)	133

Symmetry codes: (i) 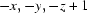; (ii) 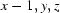. *Cg* is the centroid of the C1/C2/C7/O1/C8 furan ring.

**Fig. 1.**
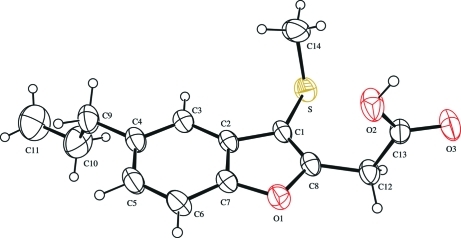
The molecular structure of the title compound with the atom numbering scheme. Displacement ellipsoids are drawn at the 50% probability level. H atoms are presented as a small spheres of arbitrary radius.

**Fig. 2.**
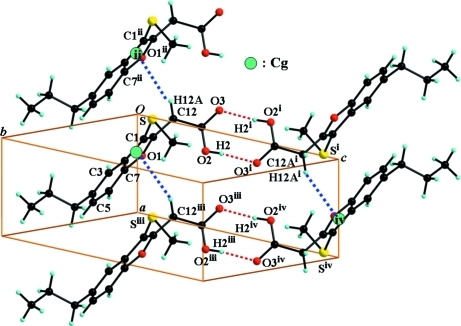
O—H···O and C—H···π interactions (dotted lines) in the title compound. Cg denotes the ring centroid. [Symmetry code: (i) -x, -y, -z+1; (ii) x-1, y, z; (iii) x+1, y, z; (iv) -x+1, -y, -z+1.]

## supplementary crystallographic information

### Comment

This work is related to our communications on the synthesis and structure of 2-(3-methylsulfanyl-1-benzofuran-2-yl)acetic acid derivatives, viz. 2-(5-ethyl-3-methylsulfanyl-1-benzofuran-2-yl)acetic acid (Seo *et al.*, 2007) and 2-(5,7-dimethyl-3-methylsulfanyl-1-benzofuran-2-yl)acetic acid (Choi *et al.*, 2008). Here we report the crystal structure of the title compound, 2-(3-methylsulfanyl-5-propyl-1-benzofuran-2-yl)acetic acid (Fig. 1).

The benzofuran unit is essentially planar, with a mean deviation of 0.005 (3) Å from the least-squares plane defined by the nine constituent atoms. In the crystal structure, the carboxyl groups are involved in intermolecular O—H···O hydrogen bonds (Fig. 2 and Table 1; symmetry code as in Fig. 2), which link the molecules into centrosymmetric dimers. These dimers are further packed into stacks along the a-axis by weak C—H···π interactions, with a C12—H12A···*Cg*^ii^ separation of 3.04 Å (Fig. 2 and Table 1; *Cg* is the centroid of the C1/C2/C7/O1/C8 furan ring, symmetry code as in Fig. 2).

### Experimental

Ethyl 2-(3-methylsulfanyl-5-propyl-1-benzofuran-2-yl)acetate (334 mg, 1.2 mmol) was added to a solution of potassium hydroxide (337 mg, 6.0 mmol) in water (20 ml) and methanol (20 ml), and the mixture was refluxed for 5h, then cooled. Water was added, and the solution was extracted with dichloromethane. The aqueous layer was acidified to pH 1 with concentrated hydrochloric acid and then extracted with chloroform, dried over magnesium sulfate, filtered and concentrated under vacuum. The residue was purified by column chromatography (ethyl acetate) to afford the title compound as a colorless solid [yield 84%, m.p. 395-396 K; *R*_f_ = 0.78 (ethyl acetate)]. Single crystals suitable for X-ray diffraction were prepared by evaporation of a solution of the title compound in diisopropyl ether at room temperature. Spectroscopic analysis: ^1^H NMR (CDCl_3_, 400 MHz) δ 0.96 (t, J = 7.32 Hz, 3H), 1.64-1.73 (m, 2H), 2.33 (s, 3H), 2.70 (t, J = 7.68 Hz, 2H), 4.03 (s, 2H), 7.13 (dd, J = 8.44 Hz and 1.44 Hz, 1H), 7.36 (d, J = 8.44 Hz, 1H), 7.43 (s, 1H), 10.02 (s, 1H); EI-MS 264 [M^+^].

### Refinement

All H atoms were geometrically positioned and refined using a riding model, with C—H = 0.93 (aromatic), 0.97 (methylene), 0.96 Å (methyl) H atoms, and O—H = 0.82 respectively, and with *U*_iso_(H) = 1.2Ueq(C) (aromatic, methylene), 1.5Ueq(C) (methyl), and 1.5Ueq(O) H atoms.

### Crystal data

**Table:**

C_14_H_16_O_3_S	*Z* = 2
*M**_r_* = 264.33	*F*(000) = 280
Triclinic, *P*1	*D*_x_ = 1.260 Mg m^−^^3^
Hall symbol: -P 1	Mo *K*α radiation, λ = 0.71069 Å
*a* = 5.1727 (6) Å	Cell parameters from 1161 reflections
*b* = 8.173 (1) Å	θ = 2.5–22.1°
*c* = 16.614 (2) Å	µ = 0.23 mm^−^^1^
α = 94.321 (2)°	*T* = 298 K
β = 95.831 (2)°	Block, colorless
γ = 91.110 (2)°	0.20 × 0.20 × 0.05 mm
*V* = 696.50 (14) Å^3^	

### Data collection

**Table:**

Bruker SMART CCD diffractometer	1425 reflections with *I* > 2σ(*I*)
Radiation source: fine-focus sealed tube	*R*_int_ = 0.049
graphite	θ_max_ = 25.0°, θ_min_ = 2.5°
Detector resolution: 10.0 pixels mm^-1^	*h* = −5→6
φ and ω scans	*k* = −9→9
3658 measured reflections	*l* = −19→14
2389 independent reflections	

### Refinement

**Table:**

Refinement on *F*^2^	Primary atom site location: structure-invariant direct methods
Least-squares matrix: full	Secondary atom site location: difference Fourier map
*R*[*F*^2^ > 2σ(*F*^2^)] = 0.059	Hydrogen site location: difference Fourier map
*wR*(*F*^2^) = 0.151	H-atom parameters constrained
*S* = 1.06	*w* = 1/[σ^2^(*F*_o_^2^) + (0.0531*P*)^2^ + 0.2044*P*] where *P* = (*F*_o_^2^ + 2*F*_c_^2^)/3
2389 reflections	(Δ/σ)_max_ < 0.001
165 parameters	Δρ_max_ = 0.21 e Å^−^^3^
0 restraints	Δρ_min_ = −0.24 e Å^−^^3^

### Special details

**Table:**

Geometry. All esds (except the esd in the dihedral angle between two l.s. planes) are estimated using the full covariance matrix. The cell esds are taken into account individually in the estimation of esds in distances, angles and torsion angles; correlations between esds in cell parameters are only used when they are defined by crystal symmetry. An approximate (isotropic) treatment of cell esds is used for estimating esds involving l.s. planes.
Refinement. Refinement of F^2^ against ALL reflections. The weighted R-factor wR and goodness of fit S are based on F^2^, conventional R-factors R are based on F, with F set to zero for negative F^2^. The threshold expression of F^2^ > 2sigma(F^2^) is used only for calculating R-factors(gt) etc. and is not relevant to the choice of reflections for refinement. R-factors based on F^2^ are statistically about twice as large as those based on F, and R- factors based on ALL data will be even larger.

### Fractional atomic coordinates and isotropic or equivalent isotropic displacement parameters (Å^2^)

**Table:**

	*x*	*y*	*z*	*U*_iso_*/*U*_eq_	
S	−0.09603 (19)	0.20796 (12)	0.21468 (7)	0.0768 (4)	
O1	0.1105 (4)	0.5482 (3)	0.39156 (13)	0.0636 (6)	
O2	0.1260 (5)	0.1733 (3)	0.45463 (16)	0.0851 (9)	
H2O	0.1607	0.0891	0.4769	0.128*	
O3	−0.2757 (4)	0.0916 (3)	0.46894 (16)	0.0785 (8)	
C1	0.0362 (6)	0.3712 (4)	0.2809 (2)	0.0520 (8)	
C2	0.2351 (6)	0.4892 (4)	0.26600 (19)	0.0516 (8)	
C3	0.3818 (6)	0.5128 (4)	0.2021 (2)	0.0591 (9)	
H3	0.3590	0.4425	0.1552	0.071*	
C4	0.5633 (6)	0.6423 (4)	0.2089 (2)	0.0627 (10)	
C5	0.5902 (7)	0.7445 (4)	0.2808 (3)	0.0711 (10)	
H5	0.7094	0.8322	0.2852	0.085*	
C6	0.4507 (7)	0.7226 (4)	0.3450 (2)	0.0708 (10)	
H6	0.4752	0.7910	0.3926	0.085*	
C7	0.2718 (6)	0.5937 (4)	0.3353 (2)	0.0556 (8)	
C8	−0.0298 (6)	0.4115 (4)	0.3551 (2)	0.0546 (8)	
C9	0.7208 (8)	0.6706 (5)	0.1400 (2)	0.0824 (12)	
H9A	0.8818	0.7278	0.1618	0.099*	
H9B	0.7653	0.5649	0.1153	0.099*	
C10	0.5895 (10)	0.7662 (7)	0.0762 (3)	0.1197 (18)	
H10A	0.5433	0.8713	0.1011	0.144*	
H10B	0.4293	0.7084	0.0542	0.144*	
C11	0.7458 (12)	0.7960 (8)	0.0082 (3)	0.146 (2)	
H11A	0.8735	0.8817	0.0253	0.219*	
H11B	0.6335	0.8284	−0.0371	0.219*	
H11C	0.8314	0.6972	−0.0075	0.219*	
C12	−0.2179 (6)	0.3359 (4)	0.4048 (2)	0.0619 (9)	
H12A	−0.3761	0.3045	0.3702	0.074*	
H12B	−0.2620	0.4176	0.4463	0.074*	
C13	−0.1180 (6)	0.1878 (4)	0.44510 (19)	0.0545 (8)	
C14	0.1689 (8)	0.0703 (5)	0.2223 (3)	0.1078 (16)	
H14A	0.3270	0.1278	0.2146	0.162*	
H14B	0.1370	−0.0192	0.1813	0.162*	
H14C	0.1847	0.0284	0.2749	0.162*	

### Atomic displacement parameters (Å^2^)

**Table:**

	*U*^11^	*U*^22^	*U*^33^	*U*^12^	*U*^13^	*U*^23^
S	0.0644 (7)	0.0620 (6)	0.1009 (8)	−0.0190 (5)	0.0062 (5)	−0.0057 (5)
O1	0.0738 (16)	0.0557 (14)	0.0620 (15)	−0.0043 (12)	0.0080 (12)	0.0099 (11)
O2	0.0554 (17)	0.0877 (18)	0.120 (2)	0.0049 (13)	0.0086 (14)	0.0595 (16)
O3	0.0556 (15)	0.0722 (16)	0.113 (2)	−0.0081 (12)	0.0101 (13)	0.0463 (15)
C1	0.0513 (19)	0.0419 (17)	0.063 (2)	−0.0047 (14)	0.0008 (16)	0.0153 (15)
C2	0.055 (2)	0.0433 (17)	0.057 (2)	−0.0015 (15)	−0.0005 (16)	0.0152 (16)
C3	0.062 (2)	0.0521 (19)	0.064 (2)	−0.0027 (16)	0.0043 (17)	0.0104 (16)
C4	0.060 (2)	0.054 (2)	0.075 (3)	−0.0056 (17)	0.0014 (18)	0.0213 (19)
C5	0.070 (2)	0.049 (2)	0.095 (3)	−0.0111 (18)	0.004 (2)	0.017 (2)
C6	0.088 (3)	0.0472 (19)	0.075 (3)	−0.0126 (19)	0.003 (2)	0.0014 (18)
C7	0.062 (2)	0.0447 (17)	0.061 (2)	−0.0007 (15)	0.0056 (17)	0.0111 (17)
C8	0.055 (2)	0.0487 (18)	0.061 (2)	−0.0023 (15)	0.0006 (16)	0.0183 (16)
C9	0.078 (3)	0.079 (3)	0.095 (3)	−0.012 (2)	0.016 (2)	0.030 (2)
C10	0.135 (4)	0.134 (4)	0.105 (4)	0.020 (3)	0.041 (3)	0.056 (3)
C11	0.190 (6)	0.151 (5)	0.107 (4)	−0.005 (5)	0.046 (4)	0.042 (4)
C12	0.055 (2)	0.060 (2)	0.075 (2)	0.0041 (16)	0.0076 (17)	0.0286 (18)
C13	0.045 (2)	0.058 (2)	0.063 (2)	0.0015 (16)	0.0080 (16)	0.0191 (16)
C14	0.080 (3)	0.059 (2)	0.183 (5)	−0.007 (2)	0.031 (3)	−0.020 (3)

### Geometric parameters (Å, °)

**Table:**

S—C1	1.746 (3)	C6—H6	0.9300
S—C14	1.791 (4)	C8—C12	1.493 (4)
O1—C7	1.381 (4)	C9—C10	1.482 (5)
O1—C8	1.385 (4)	C9—H9A	0.9700
O2—C13	1.265 (3)	C9—H9B	0.9700
O2—H2O	0.8200	C10—C11	1.487 (6)
O3—C13	1.237 (4)	C10—H10A	0.9700
C1—C8	1.332 (4)	C10—H10B	0.9700
C1—C2	1.448 (4)	C11—H11A	0.9600
C2—C7	1.376 (4)	C11—H11B	0.9600
C2—C3	1.388 (4)	C11—H11C	0.9600
C3—C4	1.392 (4)	C12—C13	1.501 (4)
C3—H3	0.9300	C12—H12A	0.9700
C4—C5	1.399 (5)	C12—H12B	0.9700
C4—C9	1.499 (5)	C14—H14A	0.9600
C5—C6	1.368 (5)	C14—H14B	0.9600
C5—H5	0.9300	C14—H14C	0.9600
C6—C7	1.377 (4)		
			
C1—S—C14	99.71 (17)	C10—C9—H9B	108.6
C7—O1—C8	105.5 (2)	C4—C9—H9B	108.6
C13—O2—H2O	109.5	H9A—C9—H9B	107.6
C8—C1—C2	106.9 (3)	C9—C10—C11	114.8 (4)
C8—C1—S	126.0 (2)	C9—C10—H10A	108.6
C2—C1—S	127.1 (3)	C11—C10—H10A	108.6
C7—C2—C3	119.6 (3)	C9—C10—H10B	108.6
C7—C2—C1	105.4 (3)	C11—C10—H10B	108.6
C3—C2—C1	135.0 (3)	H10A—C10—H10B	107.5
C2—C3—C4	119.6 (3)	C10—C11—H11A	109.5
C2—C3—H3	120.2	C10—C11—H11B	109.5
C4—C3—H3	120.2	H11A—C11—H11B	109.5
C3—C4—C5	118.0 (3)	C10—C11—H11C	109.5
C3—C4—C9	120.2 (3)	H11A—C11—H11C	109.5
C5—C4—C9	121.8 (3)	H11B—C11—H11C	109.5
C6—C5—C4	123.6 (3)	C8—C12—C13	114.0 (3)
C6—C5—H5	118.2	C8—C12—H12A	108.8
C4—C5—H5	118.2	C13—C12—H12A	108.8
C5—C6—C7	116.4 (3)	C8—C12—H12B	108.8
C5—C6—H6	121.8	C13—C12—H12B	108.8
C7—C6—H6	121.8	H12A—C12—H12B	107.7
C2—C7—C6	122.9 (3)	O3—C13—O2	124.3 (3)
C2—C7—O1	110.5 (3)	O3—C13—C12	118.8 (3)
C6—C7—O1	126.6 (3)	O2—C13—C12	116.9 (3)
C1—C8—O1	111.7 (3)	S—C14—H14A	109.5
C1—C8—C12	132.2 (3)	S—C14—H14B	109.5
O1—C8—C12	116.1 (3)	H14A—C14—H14B	109.5
C10—C9—C4	114.6 (3)	S—C14—H14C	109.5
C10—C9—H9A	108.6	H14A—C14—H14C	109.5
C4—C9—H9A	108.6	H14B—C14—H14C	109.5
			
C14—S—C1—C8	−106.1 (3)	C5—C6—C7—C2	1.1 (5)
C14—S—C1—C2	74.5 (3)	C5—C6—C7—O1	179.9 (3)
C8—C1—C2—C7	0.0 (3)	C8—O1—C7—C2	−0.2 (3)
S—C1—C2—C7	179.5 (2)	C8—O1—C7—C6	−179.0 (3)
C8—C1—C2—C3	179.1 (3)	C2—C1—C8—O1	−0.1 (3)
S—C1—C2—C3	−1.4 (5)	S—C1—C8—O1	−179.6 (2)
C7—C2—C3—C4	−0.3 (5)	C2—C1—C8—C12	−178.7 (3)
C1—C2—C3—C4	−179.4 (3)	S—C1—C8—C12	1.8 (5)
C2—C3—C4—C5	0.0 (5)	C7—O1—C8—C1	0.2 (3)
C2—C3—C4—C9	−179.2 (3)	C7—O1—C8—C12	179.0 (3)
C3—C4—C5—C6	0.9 (5)	C3—C4—C9—C10	83.8 (5)
C9—C4—C5—C6	−179.8 (3)	C5—C4—C9—C10	−95.4 (5)
C4—C5—C6—C7	−1.5 (5)	C4—C9—C10—C11	179.4 (5)
C3—C2—C7—C6	−0.3 (5)	C1—C8—C12—C13	79.2 (4)
C1—C2—C7—C6	179.0 (3)	O1—C8—C12—C13	−99.3 (3)
C3—C2—C7—O1	−179.2 (3)	C8—C12—C13—O3	−160.9 (3)
C1—C2—C7—O1	0.1 (3)	C8—C12—C13—O2	21.3 (5)

### Hydrogen-bond geometry (Å, °)

**Table:**

*D*—H···*A*	*D*—H	H···*A*	*D*···*A*	*D*—H···*A*
O2—H2O···O3^i^	0.82	1.86	2.679 (3)	174
C12—H12A···Cg^ii^	0.97	3.04	3.770 (3)	133

Symmetry codes: (i) −*x*, −*y*, −*z*+1; (ii) *x*−1, *y*, *z*.
